# The Role of Nuclear Medicine in Prostate Cancer

**DOI:** 10.3390/diagnostics15222876

**Published:** 2025-11-13

**Authors:** Isidora Grozdic Milojevic, Bogomir Milojevic, Daniel Skrijelj, Uros Bumbasirevic, Aleksandar Janicic, Boris Kajmakovic, Dragana Sobic-Saranovic, Vera Artiko, Slobodanka Beatovic

**Affiliations:** 1 Center of Nuclear Medicine and PET, University Clinical Center of Serbia, 11000 Belgrade, Serbia; 2 Faculty of Medicine, University of Belgrade, 11000 Belgrade, Serbia; 3 Clinic of Urology, University Clinical Center of Serbia, 11000 Belgrade, Serbia

**Keywords:** prostatic cancer, hybrid imaging, PET/CT, evaluation, PSMA, targeted radioligand therapy

## Abstract

**Background**: Considering the high global frequency of prostate cancer, it is necessary to know the benefits and drawbacks of numerous diagnostic and therapeutic modalities. **Methods**: In this article, we include 88 manuscripts (46/88 original studies) found on PubMed, written in English in extenso, dealing with nuclear medicine methods in patients with prostate cancer. **Results**: Choline PET/CT had low sensitivity in detecting the primary tumor. This method has been almost completely replaced by PSMA PET/CT, which is included in international guidelines and recommended for initial staging of unfavorable intermediate- to high-risk prostate cancer, the detection of recurrent disease after treatment, the evaluation of mCRPC, therapy response evaluation, and theranostics. FDG is currently used in aggressive forms of prostate cancer and as a supplement in PSMA PET/CT for patient selection for RLT. Na[^18^F]F has demonstrated satisfactory diagnostic capacity for evaluating bone loss; however, due to a lack of research, it is not recommended in international guidelines. ^18^F-Fluciclovine has lower sensitivity than [^18^F]F-PSMA-1007 for the detection of early biochemical recurrence in prostate cancer. GRPR and SSTR analogs are less frequently used but can be useful in the evaluation of rarer pathohistological types. [^99m^Tc]Tc-PSMA can be used in resource-limited settings where PET/CT is unavailable, with a lower sensitivity compared to [^18^F]F-PSMA-1007 but a higher sensitivity compared to bone scans. **Conclusions**: PSMA tracers are important tools for evaluating intermediate- and high-risk prostate cancer, with limitations in 5–10% of prostate cancers that do not express PSMA. Theranostics are increasingly incorporating PSMA.

## 1. Introduction

Prostate cancer is one of the leading health problems worldwide. Prostate cancer represents 22.6% of all diagnoses of malignant diseases in men and 10.7% of all deaths, making it the most common malignant tumor and the third leading cause of death due to malignancy in men. The incidence of prostate cancer increases with age, primarily due to cumulative somatic mutations, prolonged androgen stimulation, and chronic inflammatory changes that promote malignant transformation in prostatic epithelial cells. Thus, prostate cancer diagnosis is extremely rare in men under the age of 45, with only 6% of prostate cancer cases diagnosed in men aged 45 to 54. Higher incidence is present in men aged 55 to 69 (52%), and in men aged 70 or older (42%) [1,2].

After a digital rectal examination and evaluation of serum prostate-specific antigen (PSA) and transrectal ultrasound-guided prostate (TRUS) biopsy, a definitive diagnosis is made [1,2,3]. Based on the histopathological findings and International Society of Urological Pathology (ISUP) score, patients are classified in order to determine the best therapeutic approach [4,5].

When prostate cancer is confined to the prostate, without lymph node or distant metastases, treatment usually involves radical surgery or radiation therapy. These patients require subsequent monitoring and regular measurement of the PSA level. Although PSA remains the most established and widely used biomarker for prostate cancer, other molecular markers such as Prostate Health Index (PHI), 4Kscore, and PCA3 are being increasingly utilized to improve diagnostic specificity and risk stratification [1,2,3].

Any increase in the PSA level after radical treatment is considered a biochemical relapse of the disease, suggesting the potential occurrence of metastases and requiring additional diagnostics [3].

Specifically in the case of biochemical relapse, conventional methods such as computed tomography and bone scintigraphy are limited due to their low sensitivity in detecting metastases. While computed tomography (CT) can identify enlarged lymph nodes and visceral metastases, it has low sensitivity for detecting small or micrometastatic lymph node involvement. Its diagnostic accuracy is limited in cases of biochemical recurrence, particularly when PSA levels are below 10 ng/mL.

Magnetic resonance imaging (MRI) is currently a widely used and reliable modality for the clinical evaluation and diagnosis of prostate cancer. Due to its superior soft-tissue contrast and ability to assess both anatomical and functional characteristics, MRI has largely replaced computed tomography (CT) in local staging.

Bone scintigraphy is commonly used to detect bone metastases, but it has limited sensitivity for early or small-volume disease. It often fails to detect metastases in patients with low PSA levels (<10 ng/mL) and may produce false positives due to degenerative changes or other benign conditions. In various studies, the sensitivity of detecting LN metastases by CT scan was found to be only 16%, and the sensitivity of bone scintigraphy with ^99m^Tc-labeled compounds in detecting bone metastases was found to be 70%. The sensitivity of both methods is even lower in patients with a low PSA value [6]. The sensitivity of MRI in detecting local disease in prostate cancer varies depending on the study, but generally, it is reported to be around 85–90%. Multiparametric MRI (mpMRI) has significantly improved local staging accuracy. However, the exact sensitivity can depend on factors such as the lesion size, location, and the experience of the radiologist.

The stated shortcomings of conventional imaging methods (CT and bone scintigraphy) in prostate cancer of low stage and grade, as well as in the domain of detecting metastases in patients with biochemical relapse, have cleared a path for the development of hybrid methods: positron emission tomography with computer tomography, positron emission tomography with magnetic resonance, and single-photon tomography with computer tomography (PET/CT, PET/MR, SPECT/CT). Consequently, a wide array of different radiopharmaceuticals (mainly positron emitters) has been tested for evaluating the presence and extent of local and distant disease. Given the increasing integration of diagnostic and therapeutic nuclear medicine (theranostics), this review also discusses emerging treatment modalities alongside imaging.

## 2. PET Radiopharmaceuticals in the Evaluation of Prostatic Cancer

### 2.1. [^11^C]C-Choline or [^18^F]F-Choline

These represent the first radiopharmaceuticals used in the evaluation of prostate cancer with PET/CT. Their application is primarily driven by the enhanced uptake and turnover of phosphatidylcholine in cancer cells, which is an essential component of phospholipids in the cell membrane [6,7,8].

Different isotopes can be labeled with choline: ^11^C or ^18^F. The advantage of ^11^C-tracers over ^18^F-tracers lies in their lower excretion rate via urine, which facilitates the assessment of the prostate bed and lowers the exposure of the patient. However, their application is constrained by their short half-life of 20 min, necessitating the use of an on-site cyclotron [7,8,9]. To overcome the short half-life of ^11^C, ^18^F-labeled tracers such as [^18^F]Choline are increasingly used, offering comparable image quality with the advantage of easier distribution from central production facilities.

The sensitivity and specificity of choline PET/CT vary depending on the clinical context. Its use in the detection of primary tumors is limited due to its low sensitivity, as demonstrated in the study by Farsad et al. that showed a sensitivity of 66% and a specificity of 81% [10].

Choline PET/CT shows a good sensitivity (40.7–51.9%) and a higher specificity (98.4–99%) in the detection of lymph node metastases [11]. Compared to MRI, it has a higher sensitivity and specificity in this area [12].

The main role of choline PET/CT is to diagnose biochemical recurrence. Previous studies show that sensitivity and specificity are strongly dependent on the PSA value. Detection rates for PSA levels < 0.5 ng/mL are around 35%, while detection rates for PSA > 2.0 ng/mL increase to 80% [13]. The detection rate for patients with low PSA levels can be improved by using PSA dynamics (e.g., PSA doubling time).

**Figure 1:**
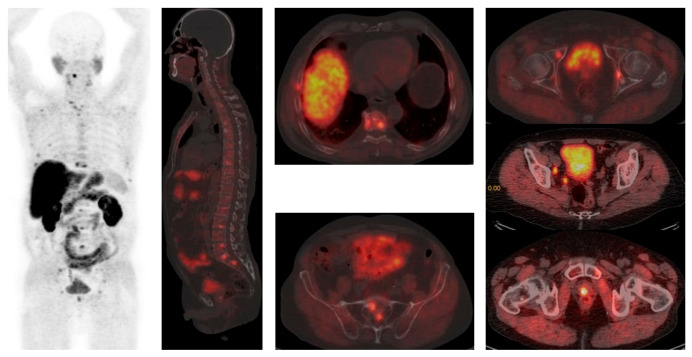
[^18^F]F-Choline PET/CT reveals widespread disease in bones (vertebrae, ribs, sacral bone) and parailiac lymph nodes. Courtesy of Ass Prof Sinisa Stojkovic, Clinical Institute of Nuclear Medicine and Thyroid Diseases, University Clinical Centre of the Republic of Srpska.

Nowadays, the use of choline PET/CT has been largely replaced by prostate-specific membrane antigen imaging (PSMA) (Figure 1). Choline PET/CT may have potential in the small subset of PSMA-negative cases.

A recent prospective, controlled, randomized trial pointed to another potential advantage of this radiopharmaceutical. Evangelista et al. stated that [^18^F]F-Choline PET/CT is more effective than conventional imaging for staging and managing intermediate- to high-risk prostate cancer. It is specifically better at detecting metastases in both the lymph nodes and bones [14].

It resulted in an approximately 8% reduction in unnecessary extended lymphadenectomies compared with contrast-enhanced CT. It also slightly affected the five-year overall survival rate, increasing it to 4%. These results support the consideration of discontinuing conventional imaging in this disease setting [14].

### 2.2. Na[^18^F]F PET/CT

Na[^18^F]F is a bone-seeking agent. After intravenous injection, it is quickly cleared from the blood pool and forms fluoroapetite crystals via chemoadsorption into the hydroxyapatite crystals in bones. The largest study that evaluated this agent was conducted by Zacho et al., who evaluated 219 scans from patients enrolled in four recent prospective trials. They included patients assessed for primary staging, biochemical recurrence, and metastatic castration-resistant prostate cancer. The interobserver agreement among trained observers in detecting bone metastases using Na[^18^F]F PET/CT in prostate cancer patients was notably high, reaching 93.6% at both the patient and lesion levels. This significant consistency underscores the reliability of this imaging modality, especially given that the number and location of bone metastases are critical factors in determining the initial course of chemotherapy/abiraterone or in offering radiotherapy, when appropriate, in men with metastatic prostate cancer. Based on previous research results, Na[^18^F]FPET/CT exhibited satisfactory diagnostic performance with a sensitivity of 86–92% and a specificity of 83–97% (positive predictive value of 70–93%, and negative predictive value of 94–96%), making it a robust diagnostic tool [15].

According to the U.S. National PET Registry study, Na[^18^F]F PET/CT changed the management plan in about 40% of patients. However, this percentage dropped to between 12% and 16% among patients who had other imaging methods scheduled [16]. In a study conducted by Gauthé et al., Na[^18^F]F PET/CT influenced management decisions in 7% of patients during initial staging [17]. Nonetheless, there is a paucity of research demonstrating that Na[^18^F]F PET/CT leads to improved patient outcomes, which may partially explain why it is not currently recommended by international guidelines for the assessment of bone metastases.

### 2.3. [^18^F]FDG PET/CT

PET with a radioactively labeled glucose analog, 2-deoxy-2-[^18^F]fluoro-D-glucose ([^18^F]FDG) enables the visualization of glucose metabolism in vivo. Fluorodeoxyglucose is rapidly and intensively taken up by most neoplastic cells due to the increased activity of glucose transporters and glycolytic metabolism.

Despite its widespread use in oncology, FDG uptake is generally low in most prostate tumors. This can be explained by the fact that most are slow-growing, metabolically inefficient, and rely on fatty acids rather than glucose for energy.

Based on previous research results, [^18^F]FDG PET/CT has been shown to be superior to [^68^Ga]Ga-PSMA-11 in rare cases of neuroendocrine differentiation where PSMA expression is low. In contrast to typical prostate adenocarcinoma, neuroendocrine prostate carcinoma (NEPC) often exhibits high glucose metabolism, making [^18^F]FDG PET/CT more effective in detecting the foci of disease. However, data on [^18^F]FDG PET/CT in NEPC are limited, and further research is needed to establish its optimal role and compare its performance with other tracers [3,18,19,20,21].

[^18^F]FDG PET/CT is currently used for advanced and aggressive disease. Increased FDG uptake is seen in aggressive, high-grade tumors and metastases, especially in PSMA-negative cases.

In metastatic castration-resistant prostate cancer (mCRPC), targeted radioligand therapy (RLT) using ^177^Lu has demonstrated significant success in treating advanced stages of the disease [22]. Metastatic castration-resistant prostate cancer patients represent a highly heterogeneous group, both in terms of prior treatment and tumor biology. Parameters which may help in predicting and optimizing response rates to RLT include [^18^F]FDG PET/CT. It appears that semiquantitative measurement of FDG uptake at baseline can be useful and can provide independent prognostic information on overall survival in mCRPC [23,24].

Lesions showing increased FDG uptake but no relevant uptake on PSMA PET (FDG+/PSMA−) were excluded for PSMA RLT in a single-center, single-arm phase 2 study with [^177^Lu]Lu-PSMA-617(LuPSMA Trial) [25]. There were encouraging results for patients receiving RLT [25], but the subjects excluded from the trial due to FDG+/PSMA− lesions had very poor OS under normal standards of care [26].

In a recent study, Michalski et al. proposed that PSMA-targeted RLT could be beneficial even in patients with PSMA-negative metastases, since it may be their last therapeutic option. Such patients underwent significantly fewer cycles of RLT either due to a missing response to PSMA-targeted RLT or due to having a more aggressive disease. However, RLT prolonged their survival compared to subjects being treated with the best standard of care [27].

**Figure 2:**
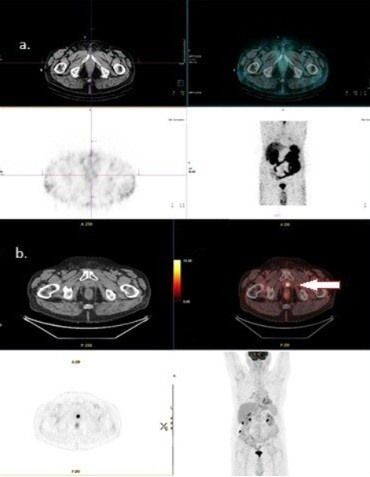
(**a**) [^99m^Tc]Tc-PSMA is negative in the same patient; (**b**) [^18^F]FDG PET/CT shows positive findings in the prostatic bed. Original clinical data from University Clinical Center of Serbia, Center of Nuclear Medicine and PET.

In summary, [^18^F]FDG PET/CT is not the imaging modality of first choice in prostate cancer, but it can supplement [^68^Ga]Ga-PSMA-11, [^18^F]^18^F-DCFPyL, and [^18^F]^18^F-JK-PSMA-7 and provide valuable information on how to select patients who are more likely to benefit from RLT; it can also provide information on prostatic cancer grade for differentiation and for assessing disease aggressiveness (Figure 2) [28].

### 2.4. PSMA-Targeting Radiopharmaceutical PET/CT

PSMA PET/CT is increasingly being integrated into the treatment of prostate cancer to improve diagnosis, treatment planning, and follow-up.

PSMA is a type II transmembrane glycoprotein with enzymatic carboxypeptidase activity. Typically, it is expressed in the cytosol of prostate tissue, but in prostate cancer cells, it becomes significantly overexpressed on the cell membrane. The level of membranous PSMA expression positively correlates with tumor grade and tends to increase in response to androgen deprivation as well as in metastatic castration-resistant cancer [3,20].

PSMA expression is not only found in the prostate. There is physiologically high expression in the proximal tubules of the kidneys, small intestine, spleen, liver, celiac ganglia, and salivary and lacrimal glands. Abnormal expression was identified in a variety of benign and malignant non-prostatic processes (fractures, fibrous dysplasia, other cancers). PSMA uptake in non-prostatic malignancies is linked to PSMA expression in tumor-related neo-vasculature. This may be relevant to its potential as a therapeutic target in non-prostate cancers. For example, it has been observed in sarcoma, breast, and ovarian cancer, where it is localized in the endothelial cells of tumor vessels [3,21].

[^68^Ga]Ga-PSMA-11 and [^18^F]F-PSMA-1007 offer high sensitivity and specificity in the detection of primary prostate cancer and metastases, especially in intermediate- and high-risk patients [3,29,30,31].

The main advantage of [^68^Ga]Ga-PSMA-11 is that it can be produced without a cyclotron, making it accessible for many nuclear medicine centers. However, compared to [^18^F]-labeled tracers, its shorter half-life and limited batch capacity can restrict its use in high-volume centers [20].

**Figure 3:**
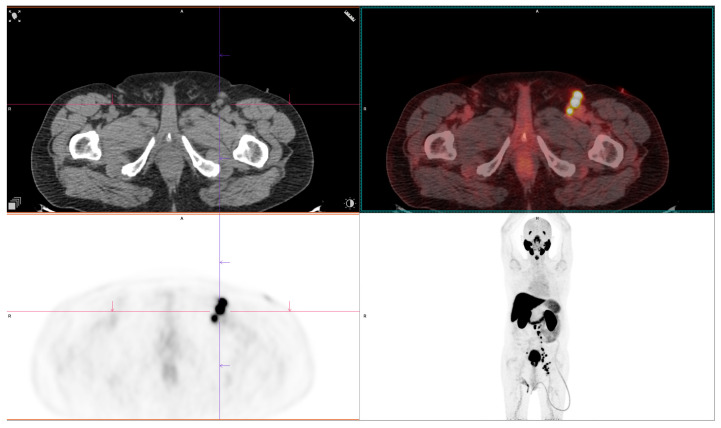
PSMA-avid lymph node metastases, highlighting the diagnostic advantage of [^68^Ga]Ga-PSMA-11 PET/CT in detecting nodal disease. Courtesy of Ass Prof Sinisa Stojkovic, Clinical Institute of Nuclear Medicine and Thyroid Diseases, University Clinical Centre of the Republic of Srpska.

With predominantly hepatobiliary excretion, [^18^F]F-PSMA-1007 has significantly lower urinary clearance compared to [^68^Ga]Ga-PSMA-11, meaning it passes through the ureters and bladder quickly and enables better visualization of changes in the prostate itself. Only 1.2% of this radiopharmaceutical is excreted through the kidneys during the first two hours after administration, while urinary excretion of [^68^Ga]Ga-PSMA-11 exceeds 10% for the same time interval (Figure 3) [20,29,30,31].

The most widely available ^18^F-based PSMA agent is [^18^F]^18^F-DCFPyL, a clinically validated, FDA-approved tracer that is highly sensitive for prostate cancer staging and recurrence detection (PSA levels ≥ 0.2 ng/mL). Its limitation is high urinary excretion, which can mask pelvic lesions [32].

[^18^F]^18^F-JK-PSMA-7 is a next-generation PSMA tracer offering high image resolution and excellent detection sensitivity even at low PSA levels (≥0.3 ng/mL). It aims to combine the image clarity and low-background uptake benefits of ^18^F-PSMA-1007 with fewer artifacts and more consistent biodistribution. It is currently under evaluation, but early studies suggest improved accuracy and fewer false-positive findings [33].

Based on international guidelines PSMA PET/CT is recommended in initial staging in high- and intermediate-risk prostate cancer patients, in evaluation of localization of biochemical recurrent and biochemical persistent prostate cancer, localization of prostate cancer which is non-metastatic according to conventional imaging (metastatic castration-resistant prostate cancer), and staging before PSMA-directed radioligand therapy [3].

### 2.5. [^18^F]F-Fluciclovine

In 2016, the Food and Drug Administration (FDA) approved [^18^F]F-Fluciclovine, a synthetic amino acid labeled with fluorine-18, for evaluating suspected recurrent prostate cancer. [^18^F]F-Fluciclovine is taken up by amino acid transporters that are overexpressed on prostate cancer cells, reflecting their increased energy and metabolic demands. The greatest strength of [^18^F]F-Fluciclovine PET/CT is its ability to localize small metastatic lymph nodes, showing superior sensitivity and specificity compared to conventional imaging modalities [34]. Nevertheless, [^18^F]F-Fluciclovine may show no uptake in lesions that appear dense on CT images (sclerotic lesions). Therefore, a bone scan or MRI may be advised. In comparison with [^18^F]F-PSMA-1007, it seems that PSMA tracers show more true-positive lesions. Previous studies have shown that there is also a trend for better inter-reader agreement for [^18^F]F-PSMA-1007 in the prostate (bed) region and lymph node metastases [34,35]. Based on the results of a prospective study, it seems that [^18^F]F-PSMA-1007 shows a superior detection rate compared to ^18^F-Fluciclovine in prostate cancer patients with early biochemical recurrence [35].

### 2.6. Other Peptides (GRPR and SSTR Analogs)

Because of the non-specificity and heterogeneous nature of prostate cancer, the diagnosis and treatment of prostate cancer is one of the most challenging tasks. Thus, precision molecular imaging has enabled us to assess the expression of specific biomarkers and dynamic changes in biomarkers in high-risk patients during disease progression and therapy.

The overexpression of tumor-specific peptide receptors—such as the gastrin-releasing peptide receptor, natriuretic peptide receptor, and somatostatin receptor—in cancer cells offers an ideal molecular basis for targeted imaging and therapeutic approaches. Radiolabeled peptides facilitate rapid clearance from the bloodstream and normal tissues, resulting in high-contrast images for positron emission tomography (PET) and single-photon emission computed tomography (SPECT). These advancements hold great potential for advancing personalized, molecular-based treatments tailored to individual patients. One of the most investigated molecular targets is the gastrin-releasing peptide receptor (GRPR), which seems to be a suitable target for diagnostics and therapy [36,37,38,39].

GRPR is a G-protein from the bombesin receptor family which is significantly overexpressed in the majority of prostate cancer cases (62–100%) but also in other cancers (breast, lung, head and neck, pancreatic) and brain malignant tumors [36,37,38,39]. Overexpression of GRPR has been found in primary prostate tumors, lymph nodes, and bone metastases. GRPR levels might decrease as the disease progresses due to its androgen-dependent expression. Several GRPR-targeting radioligands have been developed. [^68^Ga]Ga-RM2 (PET tracer) and [^111^In]In-RM2 (SPECT tracer), both showing promising preclinical and clinical results, have demonstrated their ability to safely image both primary tumors and metastatic lesions [36,37,38,39,40,41].

The heterogeneous expression of targets led to the development of a heterodimeric radiotracer with the ability to target both GRPR and PSMA. A PSMA/GRPR heterodimer [^68^Ga]Ga-DOTA-PSMA(Inhibitor)-Lys_3_-Bombesin has been clinically evaluated. While its safe toxicology and dosimetry profiles have been shown in healthy volunteers, more clinical data are still needed [36].

Furthermore, the number of neuroendocrine cells tends to rise, particularly in cases of hormonally treated and hormone-refractory (androgen-independent) prostate cancer. The lack of neuroendocrine cells in prostate tumors is associated with a better prognosis, whereas the presence of neuroendocrine differentiation in tumor samples serves as a more reliable predictor of patient survival than the Gleason score. Neuroendocrine-differentiated prostate cancer (NEPC) is an uncommon pathophysiological condition, accounting for 0.5–2.0% of all prostate cancer cases [42]. Nevertheless, focal NEPC is present in 10–100% of localized prostate adenocarcinomas; so radiolabeled somatostatin analogs can be useful for screening and treatment [42,43]. The increased availability of ^68^Ga-tracers has allowed for the development of promising PET/CT imaging for tumors with SSTRs.

## 3. SPECT Radiopharmaceuticals in Evaluation of Prostatic Cancer

### 3.1. [^99m^Tc]Tc-PSMA

**Figure 4:**
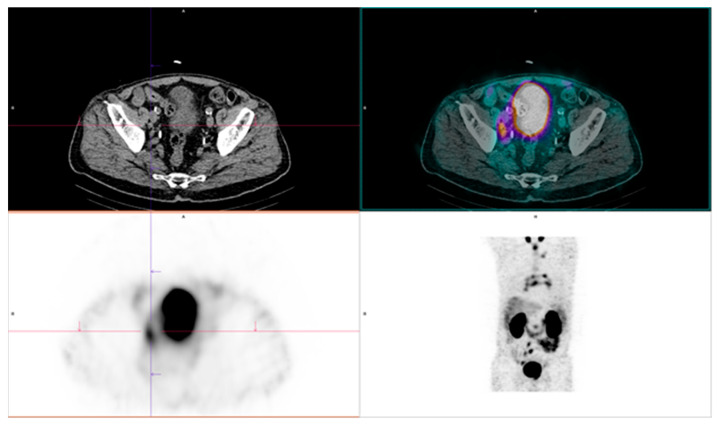
Positive findings of [^99m^Tc]Tc-PSMA. Disease is present in the right iliac lymph nodes, the retroperitoneal lymph node, and the mediastinal lymph node. Original clinical data sourced from University Clinical Center of Serbia, Center of Nuclear Medicine and PET.

Despite the advent of new PET tracers leading to a substantial decline in SPECT research efforts, ^99m^Tc continues to be the preferred radionuclide for SPECT imaging due to its optimal physical decay characteristics and easy accessibility. It is useful in the primary staging of regional and distant metastasis. Additionally, it has high diagnostic efficacy in detecting recurrent biochemical lesions after radical prostatectomy and bone metastasis [44]. With a sensitivity of 80% and a specificity of 100%, it is better than bone scans at detecting osseous lesions, with only a small amount of radiation uptake in the intestinal tract and no significant radiation uptake in other organs (Figure 4) [44,45].

Overall, while SPECT/CT has some utility, it is being increasingly replaced by PET/CT due to the latter’s superior diagnostic accuracy in both primary staging and recurrent prostate cancer. However, SPECT/CT remains widely used for detecting bone metastases in prostate cancer, especially in resource-limited settings where PSMA-targeting radiopharmaceutical PET/CT is unavailable.

### 3.2. [^99m^Tc]Tc-dyposphonate

Bone scintigraphy agents using radioactive tracer ^99m^Tc labeled with diphosphonate (most commonly methylene diphosphonate [^99m^Tc]Tc-MDP) bind to hydroxyapatite in the bones at sites of active bone formation and remodeling. It is a sensitive and widely used imaging method to detect bone metastasis in prostate cancer. While it is excellent for showing the overall extent of the disease due to its high sensitivity, it has limitations in specificity, so it cannot distinguish between cancerous lesions and benign conditions. Newer techniques like PSMA PET/CT and MRI are being used to overcome these limitations [3].

## 4. Appropriate Criteria for Use of Different Diagnostic Procedures in Evaluation of Different Stages of Prostatic Cancer

Radiology or nuclear medicine procedures are necessary in patients with prostate cancer. We can classify them based on the different stages of the disease.

### 4.1. Diagnostic Procedures in Primary Staging of Prostate Cancer

Based on EANM/SNMMI guidelines. PSMA PET/CT is recommended in the initial staging of unfavorable intermediate- to high-risk prostate cancer [3]. PSMA-targeting radiopharmaceutical ([^68^Ga]Ga-PSMA-11, [^18^F]F-PSMA-1007, [^18^F]^18^F-DCFPyL) PET/CT has 27% greater accuracy than MDCT and bone scintigraphy for detecting lymph node and distant metastases, even in early metastatic disease [3,46]. In 51% of patients, disease management is improved by detecting active disease, usually in locoregional lymph nodes or in the prostatic bead (39% or 27%, respectively). PSMA imaging has a greater ability to detect bone metastasis than bone scans, which are PSA-dependent; the sensitivity of the procedure increases with higher PSA values. It is also better at detecting other metastases (Sn 98.7%, Sp 100%) [3,46].

[^68^Ga]Ga-PSMA-11PET/CT is also beneficial since it can detect intraprostatic tumors with high specificity, aid in localization and biopsy guidance, and serve as a useful complement in inconclusive mpMRI cases [30].

Conversely, the utility of imaging in detecting lymph node metastases remains a subject of debate and ongoing development, primarily because nearly 80% of metastases occur in small, sub-centimeter lymph nodes. Consequently, the limited sensitivity of standard and hybrid imaging techniques often results in an underestimation of the true extent of disease (sensitivity of approximately 40%; specificity of around 82%) [3,37].

New ^18^F analogs are developed routinely and improve the previously stated specificity, image quality, and false-positive results of ^68^Ga tracers. Also, they can be produced at a lower cost.

### 4.2. Imaging for Biochemical Recurrence of Prostate Cancer

PET/CT is mainly used in prostate cancer for the early detection and treatment of biochemical recurrence [47,48].

Although [^18^F]F-methylcholine, [^11^C]Choline, and [^18^F]F-Fluciclovine PET/CT are utilized in recurrent prostate cancer, their effectiveness is limited at low PSA levels—the critical period when salvage therapy is most beneficial. Consequently, their use is typically reserved for cases where PSMA PET/CT results are negative, such as in neuroendocrine prostate cancer.

[^68^Ga]Ga-PSMA-11PET/CT outperforms conventional imaging modalities such as CT and bone scintigraphy, although mpMRI may show superior accuracy for local tumor staging [49,50]. Detection rates of PSMA PET/CT in recurrent prostate cancer are closely associated with serum PSA levels [47,51]. Table 1 summarizes reported sensitivities across PSA ranges, demonstrating increased detection at higher PSA values. Specificity is consistently high across all PSA values, exceeding 90%, as PSMA expression is highly specific for prostate cancer cells [51].

In addition to biochemical recurrence, [^68^Ga]Ga-PSMA-11 PET/CT is valuable for identifying oligometastatic disease, which may allow for metastasis-targeted therapy and may delay systemic treatments such as androgen deprivation therapy [52]. It also has a significant impact on clinical decision-making, with a change in treatment reported in up to 87% of cases [51].

Metastasis-directed therapy (MDT) has been evaluated in clinical trials as a treatment option for oligorecurrent prostate cancer. However, there remains a continuous evolution in the application of imaging modalities due to the ongoing development of new PSMA-targeting PET radiopharmaceuticals, which constantly reshape diagnostic and therapeutic strategies. The PRECISE-MDT study indicated that the selection of PET tracers could impact oncologic outcomes in prostate cancer patients with limited metastases undergoing metastasis-directed therapy (MDT) [53].

After propensity score matching, patients treated with [^68^Ga]Ga-PSMA-11 PET/CT-guided MDT demonstrated longer progression-free and overall survival than those treated with [^18^F]F-PSMA-1007 PET/CT-guided MDT. Furthermore, [^68^Ga]Ga-PSMA-11 exhibited better performance, with longer PFS compared to [18F]F-PSMA-1007 [51,53].

Although [^18^F]F-PSMA-1007 has been advised in some studies since it is predominantly excreted via the hepatobiliary route, caution is advised when using this radiopharmaceutical for the evaluation of bone disease [54]. In patients with biochemical recurrence of prostate cancer and PSA levels ≤ 5 ng/mL, focal bone uptake on [^18^F]F-PSMA-1007 PET/CT can be falsely positive. To prevent false bone upstaging and consequently incorrect therapy management of patients, [^18^F]F-PSMA-1007 PET/CT should be performed by experienced physicians with knowledge of unspecific bone uptake distribution patterns and characteristics [54].

### 4.3. Imaging in Metastatic Castration-Resistant Prostate Cancer (mCRPC)

PSMA PET/CT is an important imaging modality in metastatic castration-resistant prostate cancer (mCRPC), offering high sensitivity and specificity for the detection of PSMA-expressing metastatic lesions. Most patients with mCRPC develop bone metastases. In addition, visceral metastases correlate with new aggressive histological variants. PSMA-PET/CT shows a higher sensitivity in detecting lymph node, bone, and visceral metastases compared to conventional imaging [31]. It identifies PSMA-avid lesions, which are common in CRPC, and is also crucial for the selection of patients for PSMA-targeted radioligand therapy (e.g., [^177^Lu]Lu-PSMA-617) [31]. Additionally, PSMA PET/CT can reveal heterogeneity in PSMA expression, which is critical for guiding combination therapy or switching to [^18^F]FDG PET/CT for PSMA-negative, FDG-positive lesions. This dual-tracer method is particularly beneficial in advanced mCRPC cases [31].

### 4.4. Imaging in Therapy Response Evaluation

Only limited data are available on this topic. Imaging should only be used when a change in treatment plan is expected and not within 3 months of treatment to avoid a “flare” phenomenon [55,56]. Patients should be categorized as non-responders and responders (complete response, partial response, or stable disease). The use of semiquantification (SUV max) and total lesion PSMA (TL-PSMA) can quantify changes in tumor burden during therapy [54]. In addition, [^68^Ga]Ga-PSMA-11 PET/CT is used to assess the response to PSMA-targeted therapies. A reduction in PSMA uptake correlates with treatment efficacy and improved treatment outcomes. For instance, better survival results are linked to a PSMA tumor volume decrease greater than 30% following taxane-based chemotherapy [57,58,59,60,61]. Additionally, PSMA-PET enhances traditional imaging by identifying bone lesions and small-volume diseases that CT and MRI frequently overlook [60]. A limitation of PSMA PET/CT in response assessment is the persistence of PSMA expression in non-viable tumor cells, which can lead to false positive results, requiring complementary imaging or biomarkers for confirmation [51].

Emerging criteria, such as the PSMA PET Progression (PPP) criteria, standardize response assessment and increase prognostic accuracy. These criteria analyze changes in PSMA uptake and lesion size, aiding in therapeutic optimization [31].

New information about PSMA PET/CT response assessments was proposed by an expert committee (PCWG4) in 2023. It involved visual criteria that can be widely adopted without the need for proprietary software or hardware [51].

Complete response was defined as no PSMA-positive lesions. Progressive disease was defined as two new PSMA-positive lesions (no changes in intensity of uptake or SUV), with confirmation required on the 8–12-week scan. The reporter agreement of PSMA PET/CT response was substantial (0.69, 95% CI: 0.61–0.76) and displayed an almost perfect (0.90, 95% CI: 0.85–0.96) level of response (complete response, partial response, stable disease, progressive disease) and progression (progressive disease vs. non-progressive disease). Contrast-enhanced CT with RECIST is still incorporated with PSMA-targeting radiopharmaceutical PET/CT, replacing bone scans to define progression and response [51].

[^18^F]FDG PET/CT is useful in castration-resistant prostate cancer with low PSMA expression. [^18^F]FDG expression correlates with the metabolic activity of the tumor, which is a surrogate marker for tumor aggressiveness and response to systemic therapies like chemotherapy [24]. Persistent FDG uptake after systemic therapy is associated with poor prognosis [3,62].

The use of PET/CT to monitor treatment response has some limitations. The lack of standardization and tumor heterogeneity requires multimodality imaging and integration with clinical and biochemical data for a complete and reliable assessment.

## 5. Therapy of Advanced Disease

Patients with prostate cancer often advance to metastatic castration-resistant prostate cancer (mCRPC). This progression typically results from the failure of chemotherapy and hormonal castration therapies and is linked to an unfavorable prognosis [63].

In this stage, the use of drugs such as abiraterone acetate, enzalutamide, carbachol, and apalutamide is advised. Furthermore, olaparib and rucaparib are utilized to treat metastatic castration-resistant prostate cancer (mCRPC) with BRCA gene mutations. Pembrolizumab is notable as the first PD-1 inhibitor approved for prostate cancer therapy. However, the mechanisms underlying resistance to these agents remain poorly understood, and the majority of patients tend to develop either innate or acquired resistance following treatment [3,64].

Regardless of all these modalities, mCRPC continues to be incurable and will eventually progress, hence the need for more efficient agents.

Nuclear medicine therapy has long been employed for the treatment of bone metastases in prostatic cancer by using bone-seeking radiation therapy with ^223^Ra. To enhance clinical symptoms, overall survival (OS), and quality of life for patients, new drugs are currently under investigation and are rapidly advancing. Nonetheless, there remains an unmet need for effective treatments specifically targeting metastatic castration-resistant prostate cancer (mCRPC). In recent years, radionuclide-labeled therapy has achieved promising results. Targeted radioligand therapy (RLT) that specifically binds to prostate-specific membrane antigen (PSMA) has demonstrated significant potential in effectively decreasing the overall tumor burden [64,65,66].

Compared to normal prostate tissue, PSMA expression in prostate cancer tissue is elevated approximately 100 to 1000 times. This heightened expression is directly associated with androgen independence, metastasis, and disease progression, rendering PSMA an excellent target for both diagnosis and therapeutic intervention [64].

In radioligand therapy, beta-emitting isotopes ([^177^Lu]Lu-PSMA-617), which penetrate less than 2 mm into tissues, are delivered directly to PSMA-expressing tumor cells, minimizing damage to surrounding tissues. This radiopharmaceutical represents both a therapeutic and imaging agent, forming a central component of the theranostic concept that links diagnosis and targeted radioligand therapy.

PSMA-targeted RLT with ^177^Lu-therapeutical was approved by the FDA and European Medicines Agency (EMA) [67]. [^177^Lu]Lu-PSMA-617 has demonstrated strong effectiveness, safety, and ease of availability for mCRPC, along with significant clinical value and promising application prospects.

This approach has led to a significant improvement in progression-free survival and overall survival in metastatic castration-resistant prostate cancer [66]. This treatment can be combined with other therapies (e.g., androgen deprivation therapy or chemotherapy) to maximize therapeutic outcomes [68].

The VISION trial investigated this approach by enrolling patients with metastatic castration-resistant prostate cancer who had previously received at least one androgen receptor pathway inhibitor and one or two taxane-based treatments, and who exhibited PSMA-positive [^68^Ga]Ga-PSMA-11 scans [69]. Participants were randomly assigned in a 2:1 ratio to receive either [^177^Lu]Lu-PSMA-617 (7.4 GBq every 6 weeks for four to six cycles) combined with standard care permitted by the protocol, or standard care alone. The standard care regimen excluded chemotherapy, immunotherapy, 223Ra tracers, and investigational agents. A total of 581 patients received [^177^Lu]Lu-PSMA-617 alongside standard care, resulting in a significant extension of both progression-free survival and overall survival. All key secondary endpoints also showed notable improvements with [^177^Lu]Lu-PSMA-617. The incidence of adverse events of grade 3 or above was higher with [^177^Lu]Lu-PSMA-617 than without it (52.7% vs. 38.0%), but quality of life was not adversely affected. Moreover, [^177^Lu]Lu-PSMA-617 plus standard care delayed time to worsening in HRQOL and time to skeletal events compared with standard care alone [70].

The Enzap trial enrolled patients with metastatic castration-resistant prostate cancer who had not previously received docetaxel or androgen receptor pathway inhibitors and displayed PSMA-positive PET/CT scans with [^68^Ga]Ga-PSMA-11. Participants were randomly assigned in a 1:1 ratio to receive either oral enzalutamide at 160 mg daily alone or combined with adaptive dosing (two or four doses) of intravenous 75 GBq [^177^Lu]Lu-PSMA-617 every 6–8 weeks, guided by interim PSMA PET/CT assessments at week 12. The primary outcome measured was PSA progression-free survival. Results showed that adding [^177^Lu]Lu-PSMA-617 to enzalutamide significantly improved PSA progression-free survival, indicating enhanced anticancer activity in this patient population. Grade 3–5 adverse events occurred in 32 (40%) of 81 patients receiving the combination therapy and in 32 (41%) of 79 patients on enzalutamide alone [71].

The THERAP trial compared the efficacy and safety of cabazitaxel chemotherapy versus [^177^Lu]Lu-PSMA-617 in treating men with metastatic castration-resistant prostate cancer. Eligible participants had previously undergone docetaxel chemotherapy, exhibited rising prostate-specific antigen levels, and demonstrated sufficient PSMA avidity confirmed through centrally reviewed [^68^Ga]Ga-PSMA-11 and [^18^F]FDG PET/CT scans, with no discordant FDG-avid, PSMA-negative disease sites [72,73]. Patients needed adequate renal, hematologic, and hepatic function and an Eastern Cooperative Oncology Group performance status of 0–2. Prior androgen receptor-targeted therapy was permitted. Ninety-nine patients received [^177^Lu]Lu-PSMA-617, which resulted in a higher PSA response rate and fewer grade 3 or 4 adverse events [65,72].

Owing to its advantageous properties, the beta-emitting isotope ^177^Lu is currently the most widely used radionuclide in therapy. Its cytotoxic effects often achieve effective anti-tumor responses while maintaining a manageable adverse event profile [71,72].

Data from two randomized controlled studies showed that patients treated with [^177^Lu]Lu-PSMA-617 had a significantly better response compared to controls, with an odds ratio of 5.33 (95% CI: 1.24–22.90, *p* < 0.05), based on the proportion of patients experiencing a ≥50% reduction in PSA levels [73,74]. In this type of treatment, PSMA-targeting radiopharmaceutical PET/CT plays a crucial role in guiding and monitoring. PSMA-targeting radiopharmaceutical PET/CT identifies patients who are eligible for PSMA-directed radioligand therapy, as only PSMA-avid lesions are suitable for this therapy [46]. In addition, PSMA-targeting radiopharmaceutical PET/CT is used to assess treatment efficacy by evaluating changes in PSMA expression and uptake during and after therapy [27].

Targeted radioligand therapy (RLT) utilizing PSMA is an increasingly promising treatment approach for advanced metastatic castration-resistant prostate cancer (mCRPC). The fundamental concept involves “treating what you see” by pairing a diagnostic radioligand with a therapeutic counterpart, such as [^177^Lu]Lu-PSMA-617, to enable precise targeting of cancerous cells. Although this type of therapy is used in some European centers, further multicenter studies are needed to confirm the benefits of these procedures and to introduce them into wider clinical practice [75,76,77].

However, there are still non- or low responders to ^177^Lu-therapeutical. Combination radionuclide therapy, such as tandem application of [^177^Lu]Lu-PSMA-617 and [^225^Ac]Ac-PSMA-617 or ^161^Tb-therapeutical, seeks to exploit different properties (type of emission, energy, and path distance) to target heterogeneous disease and potentially enhance therapeutic outcomes [78,79,80,81,82,83,84,85,86].

## 6. Conclusions

Regardless of the different and varied radiopharmaceuticals used, PSMA imaging is becoming increasingly important in the evaluation of patients with prostate cancer. The use of PSMA is expanding, especially since it enables more sensitive staging, restaging, and selection for RLT compared to other diagnostic approaches.

The new era of nuclear medicine is theranostics, which uses radionuclide biomarker pairs that are suitable for diagnostics and radionuclide therapy. Since radioligand therapy with [^177^Lu]Lu-PSMA-617 has shown promising results worldwide, new radiopharmaceuticals labeled with PSMA (^225^Ac-therapeutical, ^161^Tb-therapeutical) are used as an alternative to decrease the negative effects of radionuclide therapy and to improve quality of life in patients with end-stage prostatic cancer.

It seems that PSMA is going to expand its usefulness in the future, as an important part of theranostics and the assessment of treatment efficacy.

## Figures and Tables

**Table 1:** Sensitivity of PSMA PET/CT for recurrent prostate cancer according to PSA level.

PSA Levels (ng/mL)	Sensitivity (%)
<0.2	33%
0.2–0.5	38–57%
0.5–1	57–74%
>1	90%

## Data Availability

Not applicable.
